# A Case of De Novo Neuroendocrine Prostate Cancer Presenting With Elevated Carcinoembryonic Antigen (CEA) Levels

**DOI:** 10.7759/cureus.114549

**Published:** 2026-08-14

**Authors:** Shunsuke Tazawa, Yusuke Ishibashi, Yasuhiro Hashimoto

**Affiliations:** 1 Urology, Misawa City Hospital, Misawa, JPN

**Keywords:** carcinoembryonic antigen (cea), de novo malignancy, neuroendocrine prostate cancer (nepc), neuron-specific enolase (nse), poor prognosis, progrp

## Abstract

Neuroendocrine prostate cancer (NEPC) is an extremely rare and highly aggressive malignancy with a poor prognosis. We present the case of a 74-year-old man who presented with weight loss and a markedly elevated carcinoembryonic antigen (CEA) level. Fluorodeoxyglucose positron emission tomography/computed tomography (FDG-PET/CT) revealed a primary prostatic mass with widespread metastases. Despite a low prostate-specific antigen (PSA) level, neuron-specific enolase (NSE) and pro-gastrin-releasing peptide (ProGRP) levels were significantly elevated. Prostate biopsy with immunohistochemical analysis confirmed neuroendocrine carcinoma with positivity for CD56 and CEA, focal positivity for synaptophysin (Syn) and chromogranin A (CgA), and negativity for PSA. Due to poor performance status, chemotherapy could not be initiated, and the patient died one month following the biopsy. De novo NEPC should be considered in patients presenting with widespread metastatic disease and markedly elevated CEA levels in the context of low PSA. Early biopsy and immunohistochemical evaluation are essential for prompt diagnosis.

## Introduction

Neuroendocrine prostate cancer (NEPC) is an aggressive, high-grade subtype of prostate cancer characterized by neuroendocrine differentiation, high metabolic activity, and an exceptionally poor prognosis [1,2]. Unlike conventional prostate adenocarcinoma, which relies heavily on androgen receptor signaling and typically produces elevated serum prostate-specific antigen (PSA) levels, NEPC often presents with low or absent PSA production despite a heavy, rapidly progressive metastatic burden. Consequently, this clinical disconnect can obscure the diagnosis and delay recognition by clinicians.

Histologically, high-grade NEPC primarily encompasses small-cell neuroendocrine carcinoma and large-cell neuroendocrine carcinoma, both of which exhibit distinct morphological features, rapid cell proliferation, and extensive necrosis [3]. Most NEPC cases encountered in clinical practice arise as therapy-related NEPC (t-NEPC) through lineage plasticity following severe selection pressure from androgen deprivation therapy (ADT) or novel androgen receptor (AR)-targeted therapies in castration-resistant prostate cancer (CRPC), occurring in up to 15%-20% of advanced cases [4-7]. In contrast, de novo NEPC, presenting at initial diagnosis without prior hormonal manipulation, is extremely rare, accounting for less than 1% of all prostate cancer diagnoses [8].

Immunohistochemically, NEPC typically exhibits strong expression of neuroendocrine markers such as chromogranin A (CgA), synaptophysin (Syn), and CD56, along with a high Ki-67 proliferation index; however, marker expression can be heterogeneous or completely negative in approximately 10% of cases [9]. This phenotypic variability, combined with atypical clinical presentations, poses substantial diagnostic challenges for both urologists and pathologists.

Here, we present a rare case of de novo NEPC (small-cell subtype) initially suspected due to a markedly elevated serum carcinoembryonic antigen (CEA) level during a workup for weight loss. At diagnosis, the patient harbored widespread lymph node, lung, liver, pleural, and bone metastases. The disease followed an exceptionally fulminant course, leading to death one month after prostate biopsy.

## Case presentation

**Figure 1:**
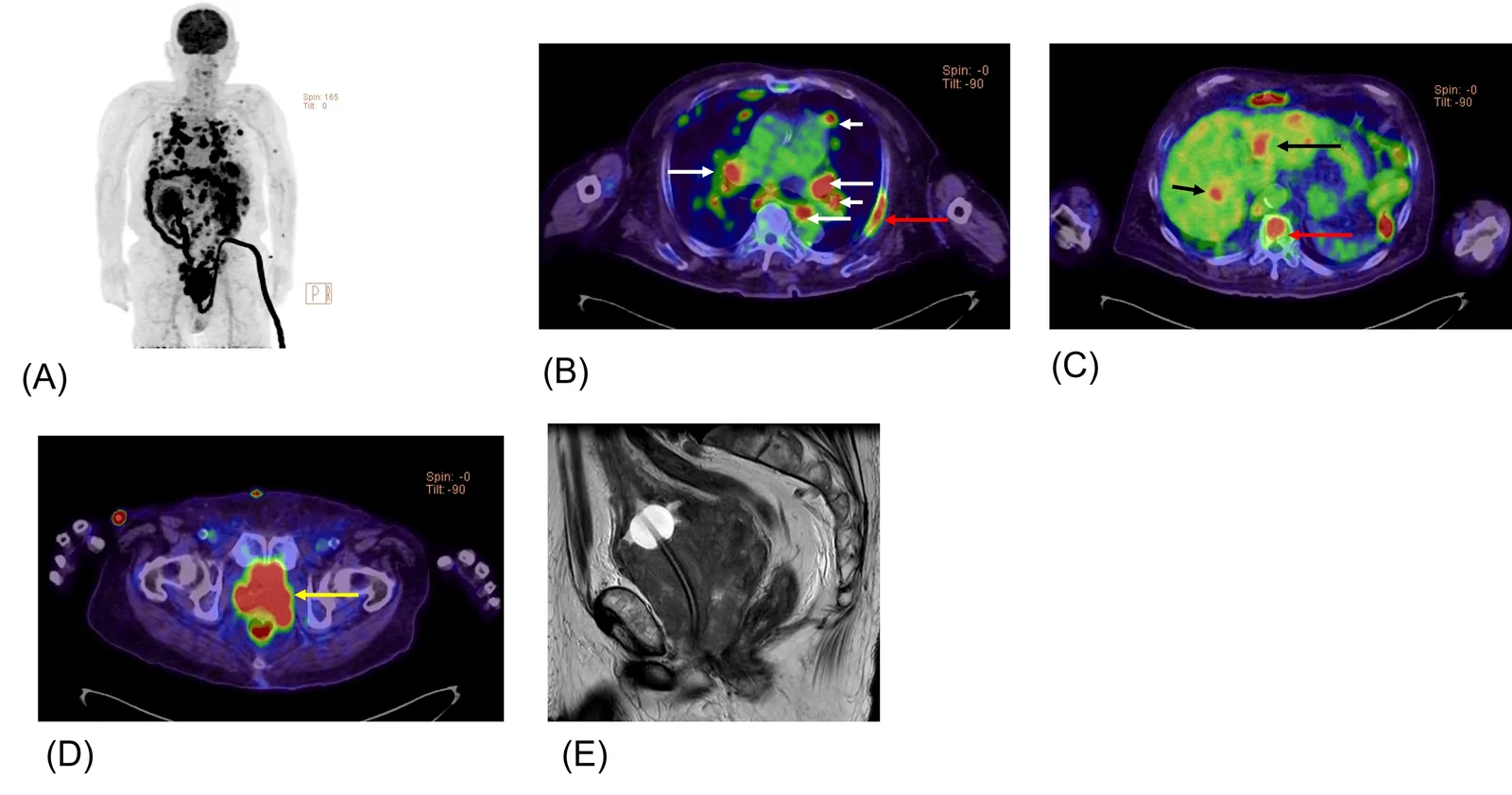
Imaging findings before prostate biopsy. A maximum-intensity-projection image from FDG-PET (A) and fused axial FDG-PET/CT images (B-D) showed intense FDG uptake in multiple lymph node metastases (B, white arrows), bone metastases (B and C, red arrows), liver metastases (C, black arrows), and the prostatic mass (D, yellow arrow). A sagittal T2-weighted magnetic resonance image (E) showed marked prostatic enlargement, with a maximum longitudinal diameter of 10 cm. FDG-PET/CT: fluorodeoxyglucose positron emission tomography/computed tomography.

A 74-year-old man presented with lower urinary tract symptoms, including progressive dysuria and nocturnal frequency, accompanied by mild lower abdominal and back discomfort. He reported a weight loss of 10 kg over the preceding three months and exhibited a reduced Eastern Cooperative Oncology Group (ECOG) performance status of 2. He was initially referred to the Department of Gastroenterology at our hospital due to a markedly elevated serum CEA level of 888.8 ng/mL noted during an outpatient workup. Digital rectal examination revealed a markedly enlarged, stony-hard, and fixed prostatic mass. Gastrointestinal endoscopy (esophagogastroduodenoscopy and colonoscopy) revealed no evidence of gastric or colorectal cancer. Fluorodeoxyglucose positron emission tomography/computed tomography (FDG-PET/CT) demonstrated a large prostatic mass, multiple lymph nodes, liver and lung metastases, pleural dissemination, multiple bone metastases, and left hydronephrosis (Figure 1). The patient was subsequently referred to the Department of Urology for further evaluation of suspected advanced prostate cancer. Laboratory tests showed a low serum PSA level (0.729 ng/mL) but markedly elevated serum neuroendocrine markers, with neuron-specific enolase (NSE) at 233.0 ng/mL and pro-gastrin-releasing peptide (ProGRP) at 1,392.7 pg/mL. Prostate magnetic resonance imaging (MRI) confirmed marked prostatic enlargement with severe bladder base compression, measuring up to 10 cm in maximum diameter (Figure 1).

**Figure 2:**
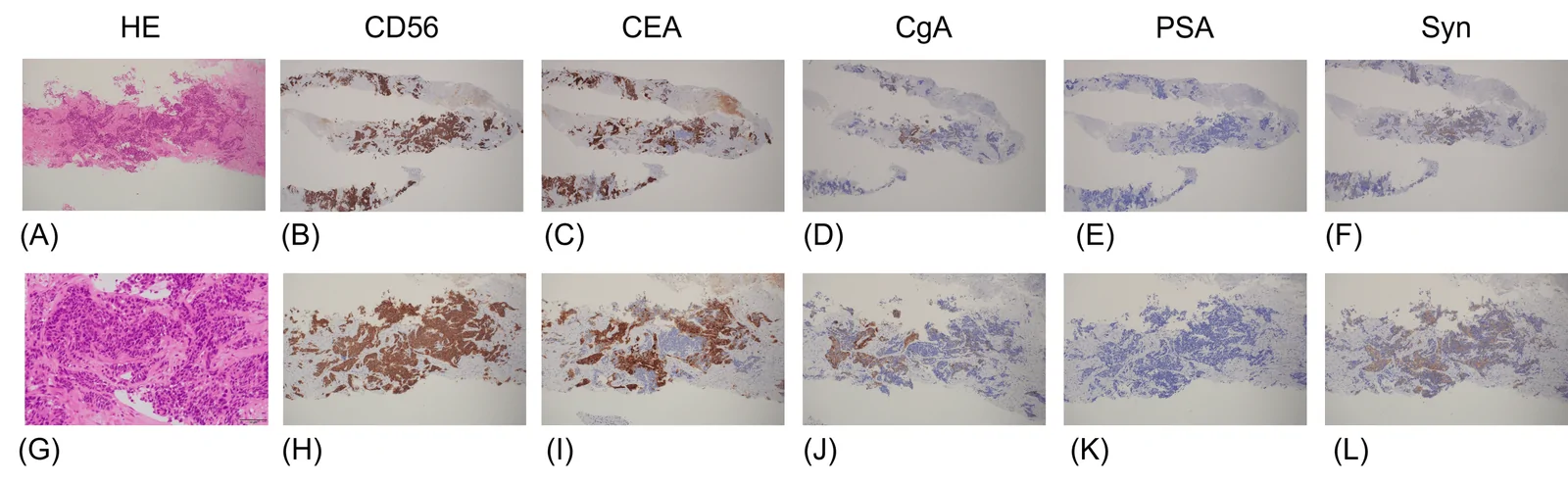
Histopathological and immunohistochemical findings of the prostate biopsy specimens. HE staining showed tumor cells with round-to-irregular nuclei arranged in alveolar clusters, with focal glandular or duct-like formation (A and G). Immunohistochemistry showed strong expression of CD56 (B and H) and CEA (C and I), focal expression of CgA (E and K) and Syn (F and L), and negative staining for PSA (F and L). Original magnification: B-F, ×40; A, H-L, ×100; G, ×400. HE: hematoxylin and eosin.

A transperineal prostate biopsy (10 cores) was performed to confirm the diagnosis. Hematoxylin and eosin (HE) staining showed all cores involved by a high-grade neuroendocrine neoplasm with features consistent with small-cell neuroendocrine carcinoma, characterized by small tumor cells with prominent nucleoli growing in alveolar clusters and focal ductal formation, accompanied by high mitotic activity and extensive geographic necrosis (Figure 2). No concurrent conventional prostate adenocarcinoma component was identified. Immunohistochemistry demonstrated strong and diffuse expression of CD56 and CEA, focal expression of Syn and CgA, a high Ki-67 labeling index exceeding 60% (data not shown), and negative staining for PSA (Figure 2). Lineage and differential markers (e.g., TTF-1, CDX2, GATA3) were evaluated to exclude primary malignancies of pulmonary, gastrointestinal, or urothelial origin.

These findings supported a diagnosis of de novo NEPC. Because of the deterioration in the patient’s general condition, chemotherapy was not administered, and the patient died one month after the prostate biopsy.

## Discussion

De novo NEPC is a rare and highly aggressive subtype of prostate cancer that exhibits neuroendocrine differentiation at initial diagnosis without prior androgen-directed therapy. It accounts for less than 1% of all prostate cancers [8] and should be distinguished from t-NEPC, which develops during the course of androgen deprivation therapy or AR-targeted therapy [8,10]. De novo NEPC frequently presents with relatively low PSA levels, visceral metastases, and rapid clinical progression [8,10].

The present case was notable for a markedly elevated serum CEA level despite a low serum PSA level, together with widespread lymph node, lung, liver, pleural, and bone metastases. CEA is widely used as a tumor marker, particularly in gastrointestinal and pulmonary malignancies, but marked CEA elevation is uncommon in conventional prostate adenocarcinoma. A recently reported patient with de novo NEPC presented with a CEA level of 1,296.5 ng/mL and a PSA level of 0.47 ng/mL; gastrointestinal endoscopy showed no evidence of gastric or colorectal cancer, and prostate biopsy demonstrated neuroendocrine carcinoma with CEA expression [2].

Similarly, the markedly elevated CEA level and weight loss in our patient initially prompted an investigation for gastrointestinal or pulmonary malignancy. However, comprehensive workup, including upper and lower endoscopy, chest/abdominal CT, and differential immunohistochemistry, ruled out primary gastrointestinal, pulmonary, and urothelial carcinomas, while imaging demonstrated a dominant mass centered in the prostate with widespread metastases. Furthermore, tumor cells showed strong CEA staining on biopsy. Although the exact contribution of the prostatic tumor to the systemic serum CEA elevation remains inferential and CEA expression alone does not conclusively prove a primary prostatic lineage, the spatial and temporal concordance between the markedly elevated serum CEA and strong intratumoral CEA expression suggests that CEA may serve as a potential supplementary marker in selected cases of de novo NEPC [2]. Therefore, de novo NEPC should be included in the differential diagnosis when a prostatic mass with extensive metastatic disease is accompanied by a low PSA level and elevated CEA or neuroendocrine marker levels.

The diagnosis of NEPC relies on the integration of characteristic histological morphology and immunohistochemical profiles. High-grade NEPC (small-cell or large-cell carcinoma) commonly expresses neuroendocrine markers such as CgA, Syn, and CD56, alongside a markedly elevated Ki-67 index, whereas PSA and androgen receptor expression are typically absent or markedly reduced [8,9]. However, immunophenotypic expression can be heterogeneous, and not all neuroendocrine markers are necessarily diffusely positive [8]. In the present case, the tumor morphology matched high-grade small-cell neuroendocrine carcinoma without an adenocarcinoma component, showing diffuse CD56 and CEA positivity, focal Syn and CgA positivity, a Ki-67 index >60%, and complete absence of PSA. These findings, together with the low PSA level and markedly elevated serum NSE and ProGRP levels, supported the final diagnosis of de novo NEPC.

Platinum-based chemotherapy, commonly combined with etoposide, is generally used for metastatic NEPC because of its clinicopathological similarity to small-cell lung cancer [10-12]. Nevertheless, treatment responses are frequently transient, and the prognosis remains dismal [4,10-12]. In the present case, the patient’s general condition deteriorated rapidly before treatment could be initiated, and chemotherapy could not be administered. He died one month after prostate biopsy. This exceptionally rapid course highlights the narrow therapeutic window in patients with advanced de novo NEPC and emphasizes the importance of prompt pathological diagnosis and early assessment of eligibility for systemic therapy.

## Conclusions

This case has several limitations. Molecular profiling was not performed; therefore, potentially relevant genomic alterations could not be evaluated. In addition, the relationship between the serum CEA elevation and tumor origin remains inferential, and CEA expression does not definitively establish a primary prostatic origin. Nevertheless, the concordance between the markedly elevated serum CEA level and strong immunohistochemical CEA expression suggests that CEA may warrant further investigation as a possible supplementary marker in selected cases of de novo NEPC. When extensive metastatic disease is accompanied by a low PSA level and elevated CEA or neuroendocrine marker levels, de novo NEPC should be considered in the differential diagnosis, and early biopsy with appropriate immunohistochemical evaluation is recommended.
